# AFB_1_ Microbial Degradation by *Bacillus subtilis* WJ6 and Its Degradation Mechanism Exploration Based on the Comparative Transcriptomics Approach

**DOI:** 10.3390/metabo13070785

**Published:** 2023-06-23

**Authors:** Peizhou Yang, Wenjing Wu, Danfeng Zhang, Lili Cao, Jieshun Cheng

**Affiliations:** Anhui Key Laboratory of Intensive Processing of Agricultural Products, School of Food and Biological Engineering, Hefei University of Technology, Hefei 230601, China; 2021171552@mail.hfut.edu.cn (W.W.); zhangdanfeng@hfut.edu.cn (D.Z.); lilycao504@hfut.edu.cn (L.C.); jieshun@hfut.edu.cn (J.C.)

**Keywords:** aflatoxin B_1_, *Bacillus subtilis*, microbial degradation, comparative transcriptomics, biological detoxification

## Abstract

Aflatoxin pollution poses great harm to human and animal health and causes huge economic losses. The biological detoxification method that utilizes microorganisms and their secreted enzymes to degrade aflatoxin has the advantages of strong specificity, high efficiency, and no pollution inflicted onto the environment. In this study, *Bacillus subtilis* WJ6 with a high efficiency in aflatoxin B_1_ degradation was screened and identified through molecular identification, physiological, and biochemical methods. The fermentation broth, cell-free supernatant, and cell suspension degraded 81.57%, 73.27%, and 8.39% of AFB_1_, respectively. The comparative transcriptomics analysis indicated that AFB_1_ led to 60 up-regulated genes and 31 down-regulated genes in *B. subtilis* WJ6. A gene ontology (GO) analysis showed that the function classifications of cell aggregation, the organizational aspect, and the structural molecule activity were all of large proportions among the up-regulated genes. The down-regulated gene expression was mainly related to the multi-organism process function under the fermentation condition. Therefore, *B. subtilis* WJ6 degraded AFB_1_ through secreted extracellular enzymes with the up-regulated genes of structural molecule activity and down-regulated genes of multi-organism process function.

## 1. Introduction

*Aspergillus flavus* and *Aspergillus parasiticus* naturally produce secondary mycotoxin metabolites, aflatoxins [1], of which aflatoxin B_1_ (AFB_1_) is the most toxic and carcinogenic due to its potential to induce liver toxicity and oxidative stress [2]. AFB_1_ pollution occurring during the processes of production, harvest, transportation, processing, and the storage of cereals, milk, and nuts usually causes serious public security problems and huge economic losses [3]. To reduce AFB_1_ contamination in food and feed, various strategies of physical, chemical, and biological approaches have been applied in the removal and degradation of AFB_1_ [4]. Although physical means (such as high temperature, ultraviolet rays, and microwave) and chemical means (such as ammoniation, neutral electric oxidation water, and sodium sulfite) have been applied in AFB_1_ degradation and removal, these two approaches have the disadvantages of an excessive energy consumption, serious nutrient loss, and even chemical residue pollution [5,6,7]. The microbial degradation and removal of AFB_1_ have the advantages of the conversion of toxins into low-toxic or nontoxic products, strong specificity, high efficiency, low energy consumption, and low toxicity without damage to nutrients [8].

The microbial degradation of AFB_1_ was carried out by microorganisms (such as *Bacillus licheniformis* [9], *Fusarium oxysporum* [10], *Levilactobacillus brevis* [11], and *Streptomyces cacaoi* [12]) and enzymes (such as catalase [13], laccase [14]). The possible mechanisms of AFB_1_ degradation and removal are: (1) adsorption by secondary metabolites *N*-acetylmuramic acid, *N*-acetylglucosamine, and peptidoglycan in the cell wall [15] and (2) degradation catalyzed by secreted extracellular or intracellular proteins [16]. The reported references with regard to the degradation of AFB_1_ mainly focused on the specific products of microorganisms, such as the protein extract of *Lactobacillus helveticus* [17], the extractable oxidase components of *Rhodococcus pyridinvorans* [18], *Bacillus licheniformis* extracellular fraction [19], and CotA laccase in *B. licheniformis* [20]. In practice, microorganisms usually simultaneously degrade and remove AFB_1_ through various enzymes for degradation and composite components for adsorption [21].

In this study, a strain of *Bacillus subtilis* WJ6 (*B. subtilis* WJ6) that degraded AFB_1_ was isolated and identified. The possible causes for AFB_1_ degradation were investigated by determining intracellular and extracellular enzymes and other components in the fermentation broth. Furthermore, the molecular mechanism of AFB_1_ degradation by *B. subtilis* WJ6 was explored based on a comparative transcriptomics approach at the mRNA expression level.

## 2. Material and Methods

### 2.1. Apparatus, Chemicals, and Kits

AFB_1_ with a purity of 99% was from Pribolab Company (China). Coumarin was from Sigma-Aldrich (Germany). The microscope with a magnification of 400 times (eyepiece of 10 times, objective lens of 40 times) was from OLYMPUS Corporation (CX33 model, Japan). A dual-beam ultraviolet–visible spectrophotometer was the TU-1901 model from Beijing Puxi General Instrument Co., Ltd. (China). High-performance liquid chromatography (HPLC) was manufactured by Waters Alliance (e2695 model, USA). Gram staining kits and other chemical reagents were from Solarbio Company (China) and Aladdin (China). The screening medium was prepared with 0.25 g KH_2_PO_4_, 0.25 g MgSO_4_ · 7H_2_O, 0.5 g KNO_3_, 0.5 g (NH_4_) _2_SO_4_, 0.005 g CaCl_2_, 0.003 g FeCl_3_ · 6H_2_O, 1 g coumarin, and 15 g agar, diluted to 1 L using double-distilled water.

### 2.2. Isolation and Molecular Identification of AFB_1_-Degrading Strain

A strain degrading AFB_1_ was screened from rotten feed contaminated with aflatoxins as follows. An amount of 5 g of sample was added to 95 mL of sterile saline, and the mixture was cultured at 37 °C with a shaking speed of 180 rpm for 2 h. Single colonies were screened on the prepared screening medium. After 12 h, single colonies were picked out for purification and further culture. In the logarithmic phase, the bacterial suspension was used for DNA extraction using Bacterial Genomic DNA Extraction Kit with the following processes. The mixture of binding solution and proteinase K was used to rapidly lyse cells and inactivate nuclease in cells. Additionally, genomic DNA was then selectively adsorbed on the silicon matrix membrane in the centrifuge column in the state of highly disordered salt. Through a series of rapid rinsing centrifugation steps and inhibitor removal, rinsing solutions were used to remove impurities such as cell metabolites and proteins. The low salt elution buffer was used to elute pure genomic DNA from the silicon matrix membrane.

The universal primers for the V3 region in the 16S-rRNA gene were F341: 5-CCTACGGGGAGGCAGCAG-3 and R518: 5-ATTACCCGGGCTGG-3. PCR amplification was carried out using the following parameters: 3 min at 95 °C initial denaturation; 15 s at 95 °C, 15 s at 56 °C, 90 s at 72 °C; 25 cycle numbers; extension time of 5 min at 72 °C. The amplified DNA was sequenced by Sangon Biotech (Shanghai, China). BLAST and MEGA11 were used for gene alignment and neighbor-joining phylogenetic tree drawing, respectively.

### 2.3. Identification Based on Physical and Chemical Properties

In this study, the physical and chemical properties, including catalase test, oxidase test, nitrate reduction test, starch hydrolysis test, arginine dihydrolase test, and V-P determination test of the bacterium were performed based on Bergey’s manual [22]. Cell staining was performed at the bacterial logarithmic phase according to the instruction of the Gram staining kit. The cell morphology was observed and recorded under the microscope at 400-fold magnification using M Shot Image Analysis System Software. The bacterial species were finally identified based on their molecular, physical, and chemical properties.

### 2.4. Bacterium Growth and Proliferation

To explore cell growth and proliferation, the concentrations of *B. subtilis* WJ6 were determined in the presence or absence of AFB_1_. The suspension samples were taken out for measurement during the 72 h fermentation. The OD_600 nm_ values of *B. subtilis* WJ6 were measured using a UV spectrophotometer [23]. In addition, the growth and proliferation of the cell were investigated under pH values of 5 to 9.

### 2.5. AFB_1_ Degradation by B. Subtilis WJ6

The efficiency of AFB_1_ degradation by *B. subtilis* WJ6 was investigated using different components of fermentation suspension. In this study, the whole suspension was divided into the following three states based on the presence of bacteria: “fermentation broth” without any treatment after fermentation, “cell-free supernatant” without strain cells after centrifugation, and “cell suspension”, only strain cells after centrifugation. After incubating *B. subtilis* WJ6 at 37 °C for 24 h under a shaking speed of 200 rpm, the cell-free supernatant and cell suspension in the fermentation broth were separated by centrifugation at 8000 rpm for 10 min at 4 °C. AFB_1_ was added to the systems of cell-free supernatant, cell suspension AFB_1_, and fermentation broth to a final concentration of 5 μg/mL. After incubating at 37 °C for 48 h under a shaking speed of 200 rpm, a 1:1 ratio of dichloromethane and suspension (*v*/*v*) was added three times to extract the AFB_1_. After drying with nitrogen, the residue was redissolved in 1 mL of acetonitrile and then filtered through a 0.22 μm filter membrane.

### 2.6. HPLC Approach Determining AFB_1_ Content

HPLC approach was used to determine AFB_1_ concentration under modified conditions based on previous reports [24]. The detailed HPLC parameters were: 43:57 of acetonitrile and water mobile phase ratio (*v*/*v*), Waters 2489 of UV detector, C18 chromatographic column of Waters XBridge (4.6 × 250 mm, 5 μm), 365 nm of detection wavelength, 1 mL/min of flow rate, 20 μL of injection volume, and 30 °C of column temperature.

### 2.7. Investigation of AFB_1_ Degradation under Different Catalytic Conditions

*B. subtilis* WJ6 cells were cultured for 48 h at 37 °C with a shaking speed of 200 rpm. The supernatant of *B. subtilis* WJ6 fermentation broth was obtained through centrifugation at 8000 rpm for 10 min at 4 °C. The effect of culture time on the degradation of AFB_1_ was investigated using the supernatant added to a final AFB_1_ concentration of 5 μg/mL during fermentation of 48 h. In addition, the effects of temperature and pH values on the AFB_1_ degradation in the supernatant were also investigated to analyze the degradation efficiency of *B. subtilis* WJ6.

### 2.8. Effects of Lignin and Coumarin on AFB_1_ Degradation of B. Subtilis WJ6

AFB_1_-degrading enzyme generally catalyzed lignin and coumarin due to their similar structures of phenolic model compounds [25,26,27]. In this study, as the sole carbon sources, lignin and coumarin were used to induce the expression of AFB_1_-degrading enzyme during the fermentation of *B. subtilis* WJ6. The effect of lignin and coumarin concentrations on the proliferation of *B. subtilis* WJ6 was investigated by the determination of OD_600 nm_ values in the broth during fermentation. After determining the effect of lignin and coumarin concentrations on cell proliferation, the lignin and coumarin concentrations with slight inhibition on *B. subtilis* WJ6 proliferation were added to the broth of *B. subtilis* WJ6 for AFB_1_ degradation. The fermentation of *B. subtilis* WJ6 was carried out at 37 °C with a shaking speed of 200 rpm in the presence of 5 g/L of lignin/coumarin, 5 g/L lignin, and 5 g/L coumarin as the sole carbon source. The broth supernatant of *B. subtilis* WJ6 was obtained after centrifugation at 8000 rpm for 10 min at 4 °C. The broth supernatant containing an initial AFB_1_ concentration of 5 μg/mL was prepared and incubated to degrade AFB_1_ at 37 °C for 48 h. The percentage of AFB_1_ degradation was calculated in the broth supernatant by the measurement of residual AFB_1_ content using the HPLC approach [24].

### 2.9. Comparative Transcriptome Analysis of B. Subtilis WJ6

*B. subtilis* WJ6 cells were cultured in LB liquid medium containing 1 μg/mL of final AFB_1_ concentration at 37 °C under a shaking speed of 200 rpm. The strain was cultured in LB liquid medium without the addition of AFB_1_ as the control. When the cell concentrations reached 1 OD_600_ value, the cells were collected by centrifugation at 8000 rpm for 10 min at 4 °C for comparative transcriptome analysis. The total RNA of *B. subtilis* WJ6 was extracted using Invitrogen TRIzol. The RNA concentration and integrity were measured using the Qubit RNA detection kit from Life Technologies Corporation (Gaithersburg, MD, USA). The RNA Seq library was constructed by Sangon Biotech (Shanghai, China). Illumina Hiseq 2500 platform was used for sequencing. FastQC was used to evaluate the quality of the original sequencing data. DESeq2 was used to analyze gene expression differences and visualize the results of gene expression differences. Differential expression gene (DEG) for analysis of gene differences, gene ontology (GO) annotate, and Kyoto Encyclopedia of Genes and Genomes (KEGG) for gene function definition were performed according to previous references [28,29].

### 2.10. Statistical Analysis

All the data above were from the experiments conducted three times. The results were expressed as mean ± standard deviation (*SD*). Software Origin 2023 was used to analyze the data and create curves and bar charts.

## 3. Results

### 3.1. Colony Isolation and Identification Based on 16S-rRNA Analysis

**Figure 1:**
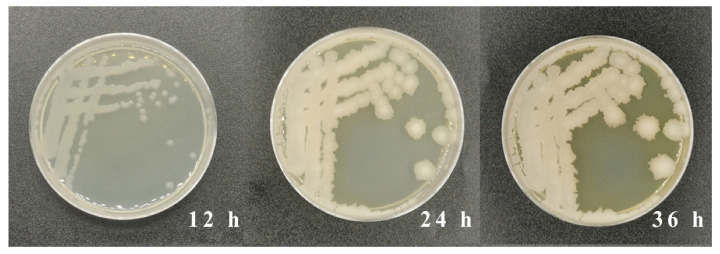
Colony morphology on LB solid media containing 10 μg/L of AFB_1_ after culture of 12, 24, and 36 h.

A strain of AFB_1_-degrading bacteria, screened from rotten feed contaminated with aflatoxins, was inoculated onto LB solid medium containing 10 μg/mL of AFB_1_. The colony morphologies were observed and recorded over a 72 h culture period. Figure 1 indicated that the diameter of the colony increased to 7 mm after 24 h of cultivation from 3 mm after 12 h of cultivation. The morphology of the colony changed to a milky-white color after a culture of 36 h from a transparent color, which was observed after a culture of 12 h. Through gene amplification and sequencing, the 16S-rRNA of the strain screened in this study had a size of 1456 bp. After sequencing, the strain was further identified using the molecular comparison on the NCBI website.

### 3.2. Colony Identification Based on Physiological and Biochemical Characteristics

**Figure 2:**
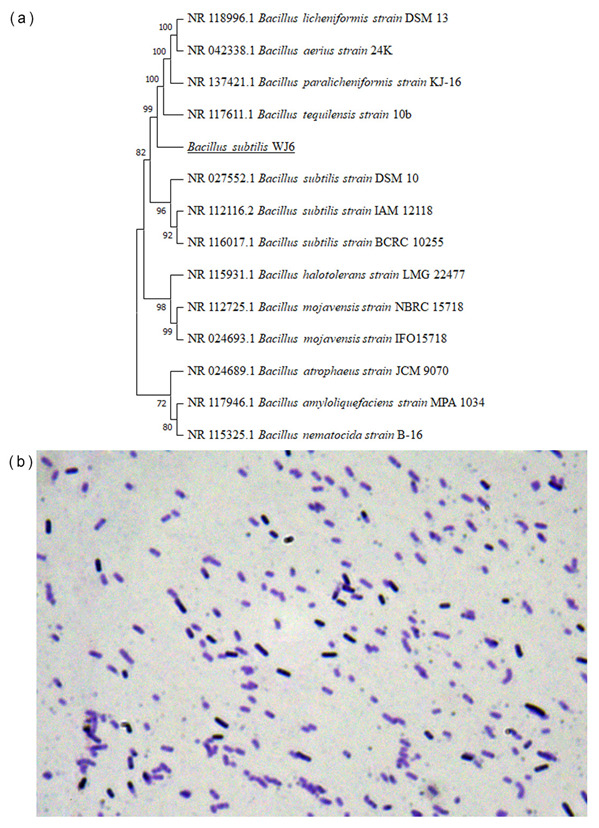
Colony genetic distance based on the 16S-rRNA sequence (**a**) and Gram staining identification of colony cells (**b**).

A phylogenetic tree was constructed using 16S-rRNA nucleotide sequences from the colony in this study and 13 other *Bacillus* strains (Figure 2a). The result showed that the genetic distance homology of 16S-rRNA of the colony was close to *B. subtilis,* while the homology of this colony had a far genetic distance from other strains, such as *B. nematocidal*, *B. amyloliquefaciens*, and *B. halotolerans.* The physiological and biochemical characteristics of the colony were performed to further identify the strain. The result of microscopy showed that the color of the colony was purple after Gram staining, which represented that the strain was Gram-positive bacteria (Figure 2b). Further, the colony was identified through physiological and biochemical experiments. The colony was aerobic, and a catalase test, oxidase, nitrate reduction to nitrite, starch hydrolysis, and V-P test results were all positive. In addition, an arginine dihydrolase test exhibited negative results. Therefore, the strain screened in this study was identified as *B. subtilis* (*B. subtilis* WJ6) based on the comprehensive analysis of the molecular identification and biological and biochemical characteristics.

### 3.3. Growth Characteristics of B. Subtilis WJ6

**Figure 3:**
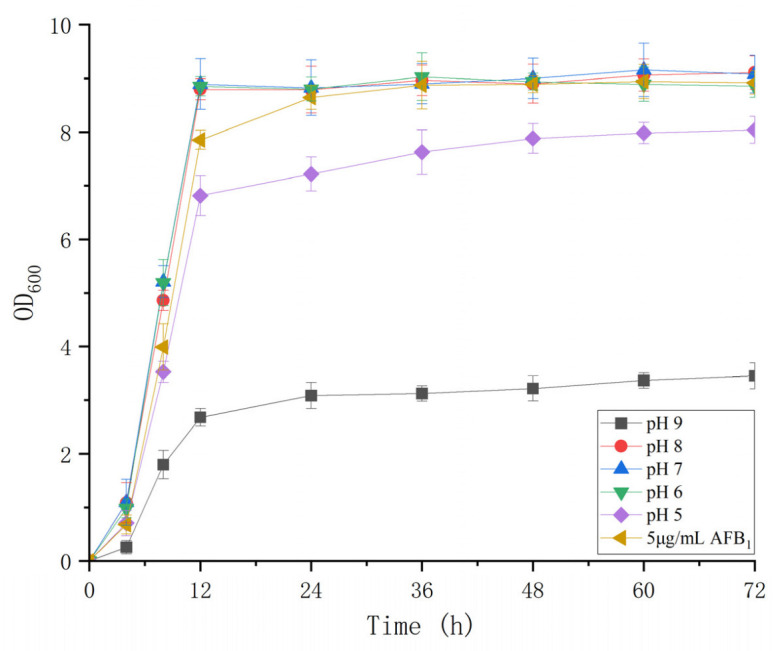
Cell proliferation of *B. subtilis* WJ6 under different pH values and 5 μg/L of AFB_1_.

The cell concentrations of *B. subtilis* WJ6 were determined under different pH values ranging from 5 to 9 (Figure 3). The results showed that *B. subtilis* WJ6 was suitable for growth and proliferation in neutral environments. Both acidic and alkaline environments inhibited the reproduction of *B. subtilis* WJ6. The OD_600 nm_ values were 3.46 and 8.04 under pH5 and 9 after a fermentation of 72 h, which were 0.38- and 0.89-fold compared to that at pH7 (9.08), respectively. Additionally, the cell concentration of *B. subtilis* WJ6 was also measured after the addition of 5 μg/mL AFB_1_. The result indicated that the addition of 5 μg/mL AFB_1_ did not affect the cell proliferation under the condition of pH7.

### 3.4. AFB_1_ Degradation Investigation of B. Subtilis WJ6 Fermentation Broth

**Figure 4:**
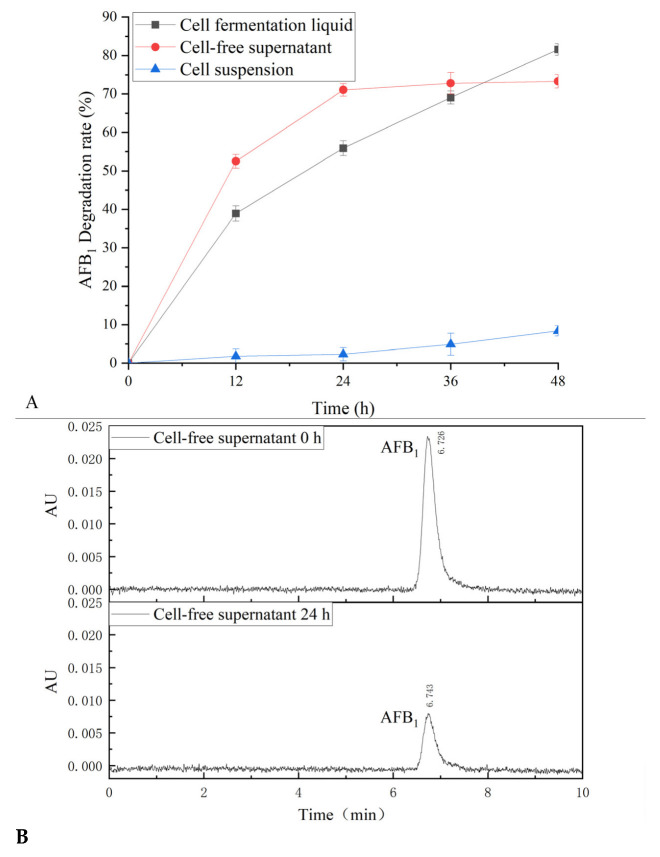
AFB_1_ degradation of *B. subtilis* WJ6 (**A**) and HPLC determination of AFB_1_ degradation (**B**).

The effect of cell fermentation liquid, cell-free supernatant, and cell suspension from *B. subtilis* WJ6 fermentation broth on the AFB_1_ degradation was investigated by measuring AFB_1_ content after catalysis (Figure 4A). The results showed that the cell fermentation liquid and cell-free supernatant had the highest AFB_1_ degradation efficiencies after a catalysis of 48 h with AFB_1_ degradation rates of 81.57% and 73.27%, respectively. Among these three groups, the cell suspension had the lowest degradation rate of 8.39%. HPLC measurement showed that the AFB_1_ content decreased with cell-free supernatant after treatment for 24 h based on the residual amount of AFB_1_ (Figure 4B). Therefore, the source of the degradation of aflatoxin could be located in the fermentation broth according to the test results.

### 3.5. Cell-Free Supernatant Affecting AFB_1_ Degradation under Different Conditions

**Figure 5:**
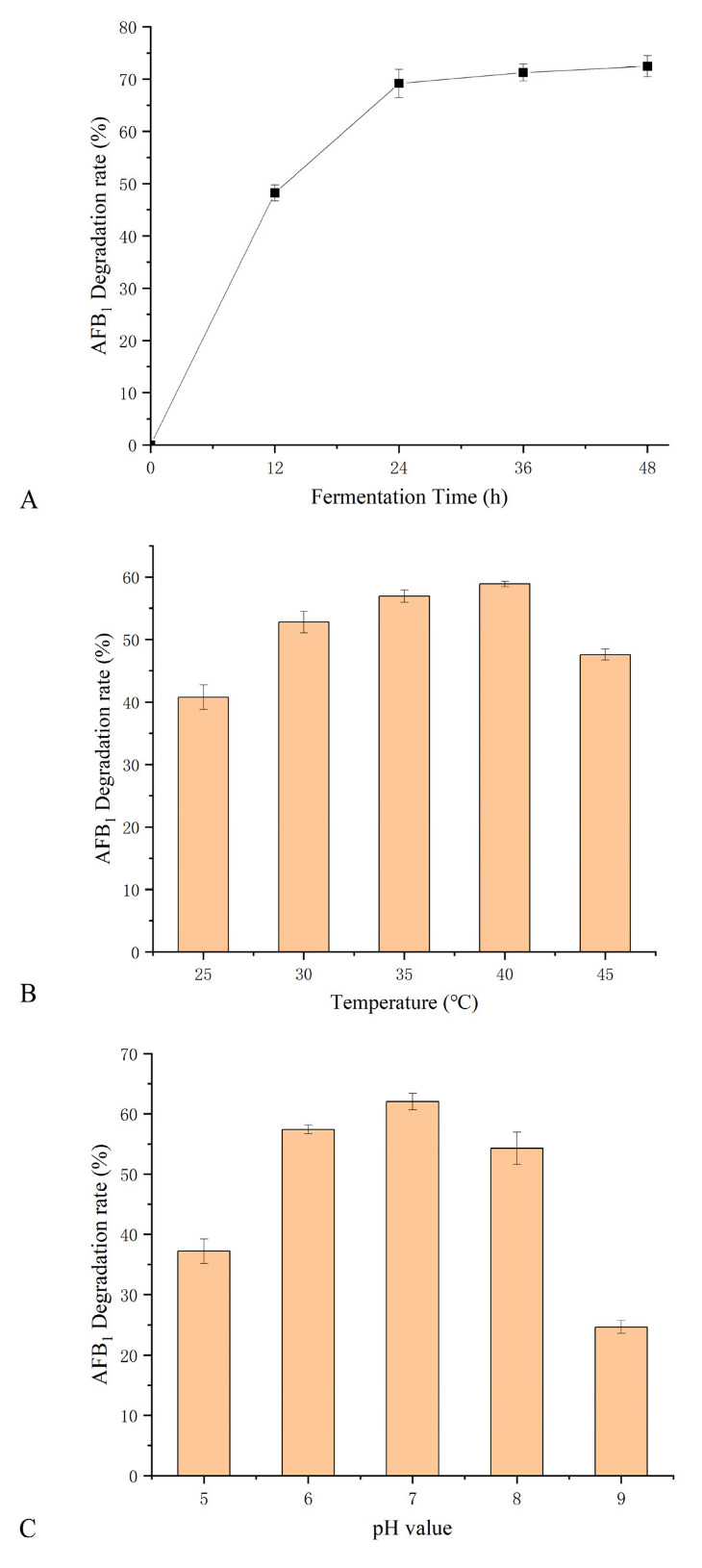
Effect of fermentation time (**A**), temperature (**B**), and pH values (**C**) on AFB_1_ degradation in cell-free supernatant from *B. subtilis* WJ6.

The effect of culture time, pH, and temperature on AFB_1_ degradation was investigated in *B. subtilis* WJ6 cell-free supernatant by measuring AFB_1_ concentration (Figure 5). The results indicated that the AFB_1_ degradation rate of cell-free supernatant was close to the highest when the fermentation time was 24 h during the period of 48 h (Figure 5A). The effect of temperatures on AFB_1_ degradation was investigated using *B. subtilis* WJ6 cell-free supernatant as a catalyst (Figure 5B). The result showed that under the condition of 40 °C the AFB_1_ degradation rate was 58.5%, which was the highest among the tested temperatures of 25, 30, 35, 40, and 45 °C. Additionally, the effect of pH values on the AFB_1_ degradation was also investigated in the cell-free supernatant from the fermentation broth of *B. subtilis* WJ6 (Figure 5C). The AFB_1_ degradation rate reached 63.27% under the pH value of 7, which was the highest among the values of 5–9. The result indicated that cell-free supernatant under pH-neutral conditions had the highest catalytic degradation efficiency of AFB_1_. Therefore, under the conditions of 24 h fermentation, 40 °C, and pH 7, *B. subtilis* WJ6 cell-free supernatant possessed the highest AFB_1_ degradation efficiency.

### 3.6. Effect of Lignin and Coumarin Concentrations on the Cell Proliferation of B. Subtilis WJ6

**Figure 6:**
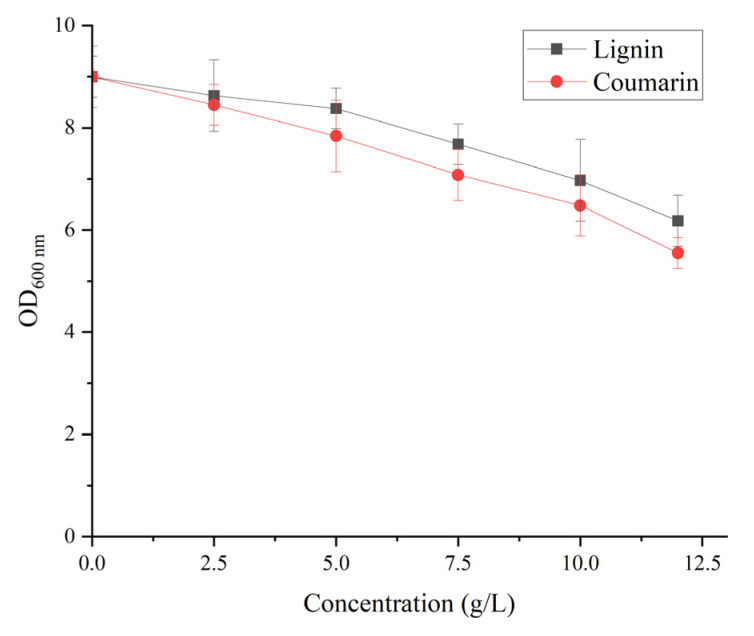
Effect of different lignin and coumarin concentrations on the cell proliferation of *B. subtilis* WJ6.

The effect of lignin and coumarin concentrations on the cell proliferation of *B. subtilis* WJ6 was investigated by measuring the cell density after a fermentation of 36 h (Figure 6). The result indicated that the concentrations of lignin and coumarin from 0–5 g/L had a slight inhibitory effect on the cell proliferation of *B. subtilis* WJ6. OD_600 nm_ values decreased to 8.4 and 7.8 from the initial OD_600 nm_ value of 9 using 5 g/L of lignin and coumarin as the sole carbon source, respectively. While the lignin and coumarin concentrations of 5–10 g/L could remarkably enhance the inhibitory effect on the cell production of *B. subtilis* WJ6. OD_600 nm_ values decreased to 7 and 6.5 using 10 g/L of lignin and coumarin, respectively. Thus, after a comprehensive consideration of the dose and its impact on cell growth, 5 g/L of lignin and coumarin could be suitable conditions to induce the expression of AFB_1_-degrading enzymes.

### 3.7. AFB_1_ Degradation of B. Subtilis WJ6 in the Presence of Lignin and Coumarin

**Figure 7:**
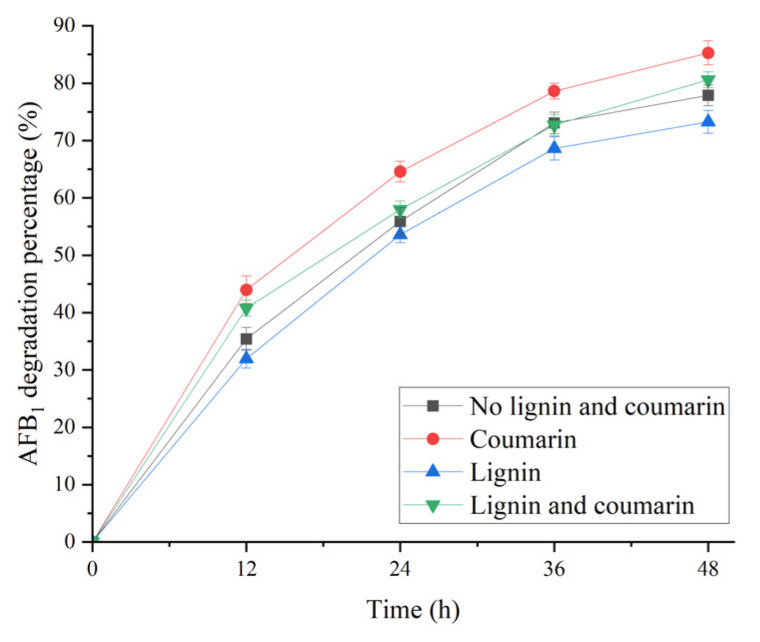
Effect of treatment time on AFB_1_ degradation percentages of *B. subtilis* WJ6 in the presence of 5 g/L lignin and coumarin.

In this study, the effect of 5 g/L lignin and coumarin on AFB_1_ degradation in the broth from *B. subtilis* WJ6 was investigated by measuring the residual content of AFB_1_ after catalysis (Figure 7). The results indicated that 5 g/L coumarin treatment had the highest degradation percentage of AFB_1_ (85.3%) among the four groups, which was 1.1-fold of the control (77.8%). In addition, 5 g/L lignin treatment resulted in the lowest degradation percentage of AFB_1_ (73.3%), which was 0.9-fold of the control (without the addition of coumarin and lignin). Therefore, coumarin had more AFB_1_ degradation capability than lignin in the broth supernatant of *B. subtilis* WJ6.

### 3.8. Differentially Expressed Genes (DEGs)

**Figure 8:**
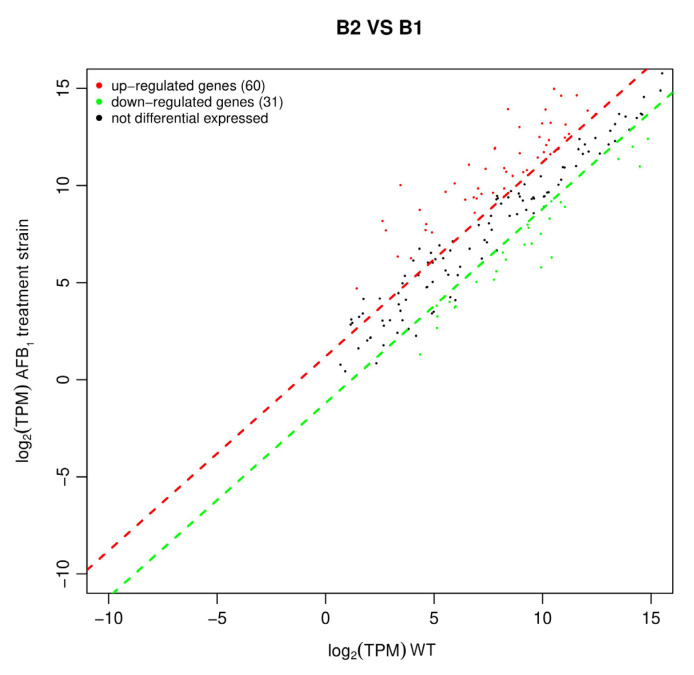
Up-regulated and down-regulated genes of *B. subtilis* WJ6 based on DEGs.

DEGs data had been submitted in Supplementary File S1. The scatter diagram of differential gene expression was drawn based on the transcriptomics sequencing (Figure 8). The results showed that *B. subtilis* WJ6 in the presence of AFB_1_ caused 91 differentially expressed genes, including 60 up-regulated genes and 31 down-regulated genes. Thus, AFB_1_ in the fermentation broth resulted in more up-regulated genes than down-regulated genes in *B. subtilis* WJ6 at the transcriptome level.

### 3.9. GO Annotation of Differentially Expressed Genes

**Figure 9:**
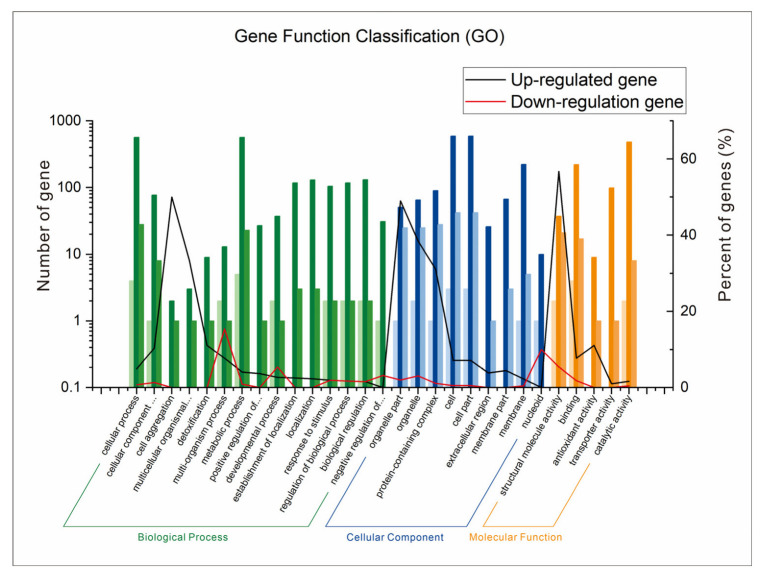
GO annotation analysis of differentially expressed genes of *B. subtilis* WJ6.

The GO annotation analysis of differentially expressed genes was performed using data from transcriptomics sequencing (Figure 9). A total of 29 gene function classifications were divided into three gene function classification groups: biological processes (15 gene classifications), cell components (9 gene classifications), and molecular functions (5 gene classifications). Among the up-regulated genes, in each classification, 50%, 49%, and 56.8% of DEGs participated in cell aggregation, the organizational part, and structural molecule activity, respectively. In addition, among the down-regulated genes, 15.4% of DEGs participated in the multi-organism process. Therefore, AFB_1_ could result in up-regulated gene expression related to functions of cell aggregation, the organizational part, and structural molecule activity, and down-regulated gene expression related to the multi-organism process function under the fermentation condition.

### 3.10. KEGG Enrichment Analysis Based on Differential Gene Function

**Figure 10:**
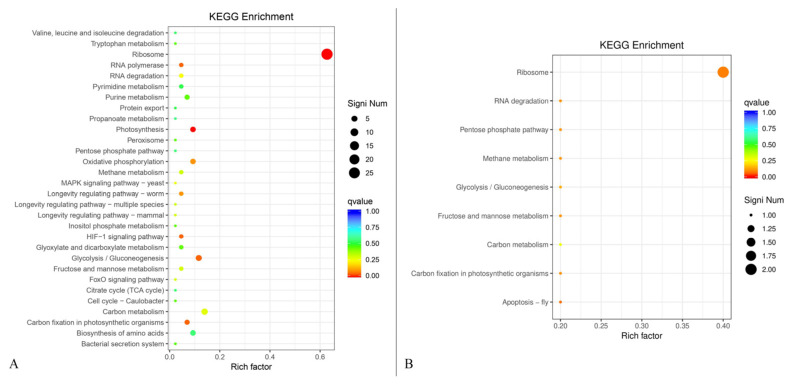
Up-regulated genes (**A**) and up-regulated genes (**B**) of *B. subtilis* WJ6 based on KEGG enrichment analysis.

KEGG enrichment was analyzed based on the differential gene function using the cluster profiler plot analysis approach (Figure 10). KEGG enrichment of the up-regulated genes showed that a total of 30 gene function classifications were given. The up-regulated genes related to ribosome had the highest proportion of differential genes (60.4%) with 29 gene numbers (Figure 10A). All the proportions of differential genes in 29 other differential function classifications were less than 20%. In addition, the KEGG enrichment of the down-regulated genes was also analyzed (Figure 10B). A total of nine gene function classifications were used to construct the KEGG enrichment map. The down-regulated genes related to ribosome had the highest rich factor of 0.4 among these function classifications. Thus, AFB_1_ caused more up-regulated genes of *B. subtilis* WJ6 than down-regulated genes based on KEGG enrichment. 

## 4. Discussions

AFB_1_, a natural toxin produced by food-contaminant fungi, is a threat to human and animal health. The microbial degradation of AFB_1_ was achieved via biological adsorption, living organisms, supernatants, and purified enzyme approaches [30]. Previous references showed that AFB_1_ could be degraded by microorganisms via the enzymes secreted in the fermentation supernatant (Table 1). Multiple strains such as *Trichoderma reesei* [31], *Stenotrophomonas acidoaminiphila* [32], *Lactobacillus plantarum* [33], and *Cerrena unicolor* [34] possessed the capability of AFB_1_ degradation by extracellular enzyme expression in a liquid medium. The percentages of AFB_1_ degradation by different strains varied from 46.9% to 98.56% under the conditions of different treatment time and initial AFB_1_ concentrations. *Bacillus* strains possessed a strong capability of AFB_1_ degradation in the fermentation medium [35,36,37]. In this study, *B. subtilis* WJ6 could degrade 81.57% of AFB_1_ from the initial concentration of 5 μg/mL after treatment for 48 h through an extracellular enzyme in a liquid medium. The high AFB_1_ degradation rate generally needed a long treatment time and/or initial AFB_1_ concentration. For example, *Stenotrophomonas* sp. degraded 91.2% of AFB_1_ with a low initial AFB_1_ concentration of 0.047 μg/mL by a combination of enzymes and oxides [32]. In addition, *C. unicolor* was able to degrade 98.56% of AFB_1_ after a period of 14 d [34]. *B. subtilis* WJ6 possessed a higher efficiency of AFB_1_ degradation with a higher initial concentration of AFB_1_ and shorter catalytic time compared with the reported strains [35,38]. Additionally, this study also revealed the mechanism based on the up-regulated genes of structural molecule activity and the down-regulated genes of multi-organism process function. Therefore, *B. subtilis* WJ6 still had an advantage in AFB_1_ degradation based on the comprehensive comparison of substrate concentration, catalytic time, and degradation efficiency. 

Glycolysis and gluconeogenesis path analyses were performed using the DEGs data based on the transcriptomics (Supplementary File S2). Enzymes participating in the glycolysis/glucose neogenesis pathway exhibited significant overexpression. In the pentose phosphate pathway, the conversion of glyceraldehyde-3-P from *D*-fructose-1,6-P_2_ via glycerone-P and glyceraldehyde-3-P, represented significant overexpression. In addition, the enzyme for the conversion of glycerate-2-P and phosphoenolpyruvate also displayed significant overexpression. Therefore, gene expression related to AFB_1_ degradation could involve in the pentose phosphate pathway. The possible limitations of this study lacked the determination of specific AFB_1_ decomposition enzymes and the feasibility analysis of the multi-enzyme degradation of AFB_1_ using the synergistic approach. In addition, artificial intelligence and machine learning would be used to modify the AFB_1_-degrading enzyme with high activity and specificity, which was a key direction of AFB_1_ degradation in the future.

## 5. Conclusions

AFB_1_ microbial degradation is a promising field due to its guarantee of nutritional values, reduction in AFB_1_ without residual toxicity, and no modification of food or feed properties. In this study, *B. subtilis* WJ6, with the capability of AFB_1_ degradation, was identified using molecular, physiological, and biochemical properties. The cell-free supernatant after fermentation could effectively degrade AFB_1_ under the conditions of 40 °C and pH 7. A percentage of 81.57% was degraded in the cell-free supernatant of *B. subtilis* WJ6 with an initial AFB_1_ concentration of 5 μg/mL after treatment for 48 h. As the sole carbon source, coumarin had a higher AFB_1_-degrading capability than lignin in the broth from *B. subtilis* WJ6. Further, the result of comparative transcriptomics analysis indicated that AFB_1_ caused 60 up-regulated genes and 31 down-regulated genes in *B. subtilis* WJ6. GO annotation analysis showed that 50%, 49%, and 56.8% of DEGs participated in cell aggregation, the organizational part, and structural molecule activity among the up-regulated genes, respectively. KEGG analysis showed that the up-regulated genes related to ribosome had the highest proportion of differential genes (60.4%) with 29 gene numbers. AFB_1_-degrading *B. subtilis* WJ6 degraded AFB_1_ through the extracellular enzymes secreted in cell-free supernatant. 

## Figures and Tables

**Table 1:** Microbial AFB_1_ degradation efficiency and possible degradation mechanisms.

Strains	AFB_1_ Degradation, Initial Concentration, Catalytic Time	AFB_1_ Degradation Mechanisms
*T. reesei* [31]	87.6%, 10 μg/kg, 120 h	Enzyme in liquid medium
*S. acidoaminiphila* [32]	91.2%, 0.047 μg/mL, 24 h	Enzymes/oxides combination
*A. niger* [39]	88.59%, 2 μg/mL, 72 h	Intracellular enzymes
*C. unicolor* [34]	98.56%, 5 μg/mL, 14 d	Lac 2
*L. plantarum* [33]	84.6%, 0.15 μg/mL, 24 h	Fermentation supernatant
*B. lysinibacillus* [35]	61.3%, 5 μg/kg, 48 h	In liquid medium
*B. sporosarcina* [35]	46.9%, 5 μg/kg, 48 h	In liquid medium
*B. staphylococcus* [36]	56.8%, 5 μg/kg, 48 h	In liquid medium
*B. amyloliquefaciens* [37]	84%, 5 μg/mL, 96 h	Extracellular enzyme
*B. subtilis* [38]	74%, 5 μg/kg, 7 d	In liquid medium
*B. subtilis*, this study	81.57%, 5 μg/mL, 48 h	Extracellular enzyme in cell-free supernatant

## Data Availability

All the data generated in the study were included in the present manuscript.

## Supplementary Materials

The following supporting information can be downloaded at: https://www.mdpi.com/article/10.3390/metabo13070785/s1, The data of DEGs and glycolysis and gluconeogenesis path analysis using the DEGs data based on the transcriptomics have been listed in Supplementary Files S1 and S2, respectively.
