# Supplementary figures and images for: AFB1 Microbial Degradation by Bacillus subtilis WJ6 and Its Degradation Mechanism Exploration Based on the Comparative Transcriptomics Approach
Source: Metabolites. 2023 Jun 23;13(7):785. doi: 10.3390/metabo13070785 (PMC10385142; doi:10.3390/metabo13070785)

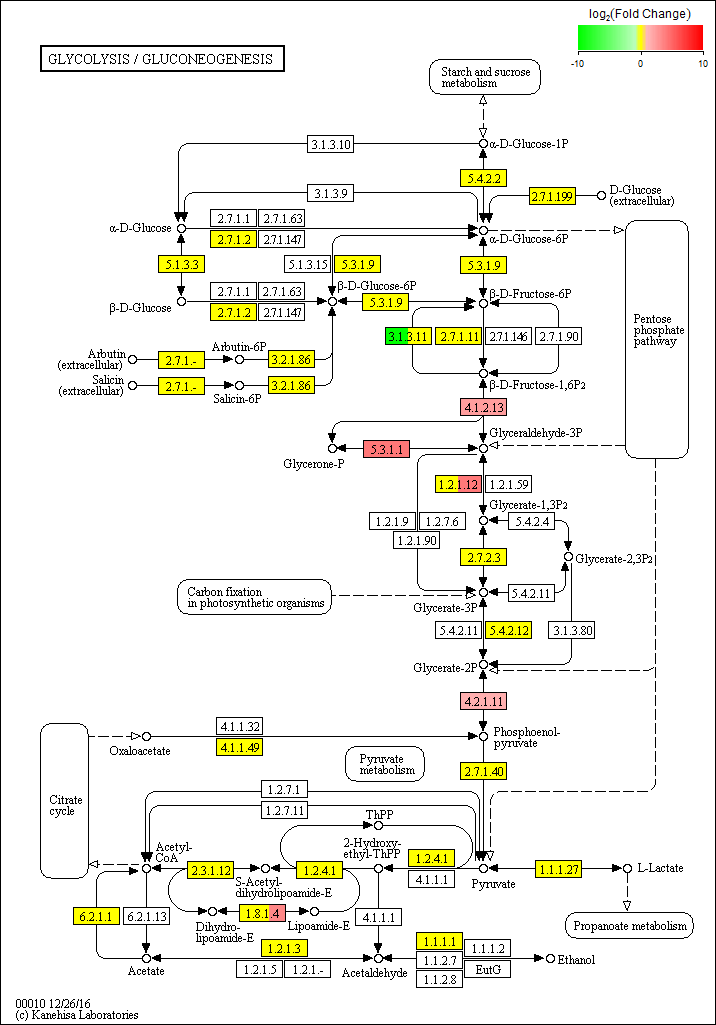

Supplement: Supplementary file 1 [file metabolites-13-00785-s001.zip › Supplementary File 2.png]
